# Obesity in Total Hip Arthroplasty: Demographic Disparities and Outcome Incongruities

**DOI:** 10.7759/cureus.7955

**Published:** 2020-05-04

**Authors:** Wayne A Wilkie, Ethan A Remily, Nequesha S Mohamed, Scott McDermott, Bernard Shalit, Andrew Baird, Kenneth Brand, James Nace, Ronald Delanois

**Affiliations:** 1 Orthopaedics, Lifebridge Health-Rubin Institute for Advanced Orthopedics, Baltimore, USA

**Keywords:** total hip arthroplasty, obesity, morbid obesity, utilization, tha

## Abstract

Introduction

As morbid obesity disproportionately affects minorities and those of lower socioeconomic status, body mass index (BMI) restrictions on total hip arthroplasty (THA) may harm populations already facing disparities in care. Therefore, this study analyzed demographics and outcomes in morbidly obese primary THA patients.

Methods

The National Inpatient Sample was queried for THAs performed between 2009 and 2016. Of 2,676,086 patients identified, 453,250 had a BMI over 25 kg/m^2^. Patients were stratified by BMI into overweight (BMI=25.0-29.9 kg/m^2^), non-morbidly obese (BMI=30.0-40.0 kg/m^2^), and morbidly obese (BMI>40.1 kg/m^2^). Patient demographics (age, sex, race, insurance, income, and Charlson Comorbidity Index) and outcomes (length of stay [LOS], mortality, disposition, complications, charges, and costs) were assessed. Categorical and continuous data were analyzed with chi-square analyses and one-way analyses of variance, respectively.

Results

The number of overweight, non-morbidly obese, and morbidly obese patients increased by 299.0%, 109.3%, and 90.9%, respectively, between 2009 and 2016 (p<0.001). Morbidly obese patients were younger than non-morbidly obese and overweight patients (p<0.001) and had a higher proportion of females (p<0.001) and black patients (p<0.001). Morbidly obese patients most frequently used Medicaid and private insurance (p<0.001). Morbidly obese patients demonstrated a longer LOS, a higher mortality rate, a lower rate of home discharges and the most complications (all, p<0.001).

Conclusion

These results reflect the worsening obesity epidemic and may be useful in counseling preoperative weight loss to morbidly obese patients to reduce mortality and complications.

## Introduction

As the annual volume of total hip arthroplasty (THA) in the United States approaches 635,000 procedures by 2030, the Center for Medicare and Medicaid Services (CMS) has targeted this expensive surgery with alternative payment models that reduce cost by shifting the financial burden of readmissions and complications onto the hospital systems and providers [1,2]. Nevertheless, the growing obesity epidemic and concurrent demand for THA by obese patients has complicated the selection process for hospital systems. To avoid financial penalties from the increased prevalence of deep vein thromboses, cardiac arrests, and infections associated with an elevated body mass index (BMI), many hospital systems have instituted BMI cut offs that prevent morbidly obese patients from undergoing THA until their weight is optimized [3-6]. However, since the obesity epidemic disproportionately affects populations already experiencing healthcare disparities, BMI restrictions may unintentionally alienate those with the greatest need by placing another barrier to care.

Obesity is undoubtedly a lifestyle disease; yet, there is ample evidence that social determinants of health are key risk factors that may inhibit patients from losing weight before surgery. For instance, people without college educations are twice as likely to be obese than their college educated counterparts [7]. Additionally, lower socioeconomic status is associated with higher rates of obesity. Interestingly, one study found that simply moving out of a highly impoverished neighborhood significantly reduced the odds of developing this disease [7]. Finally, ethnicity is associated with obesity, as African American and Hispanic communities face disproportionately higher rates of obesity than non-Hispanic Caucasians [8,9]. This alienation is concerning, as a cost-benefit analysis has suggested the benefits of THA in obese patients of all severities outweigh the associated risks and costs [10]. Given these findings, when surgeons render obesity down to a simple modifiable risk factor that must be optimized before THA, they risk placing undue burdens upon those with the least ability to change their situation.

To ensure safe, cost-effective surgery is performed without alienating those in greatest need, orthopedic surgeons may benefit from understanding the demographics, cost, and outcomes associated with obese patients. Unfortunately, there is a dearth of national studies that include an in-depth stratification of BMI. Therefore, this study utilizes a large national database to assess the effect of THA in patients with higher BMIs. Specifically, we evaluate the incidence, demographics, costs, and outcomes in non-obese (BMI<25.0 kg/m^2^), overweight (BMI=25.0-29.9 kg/m^2^), non-morbidly obese (BMI=30.0-39.9 kg/m^2^), and morbidly obese (BMI>40.0 kg/m^2^) THA patients.

## Materials and methods

Data source

The National Inpatient Sample (NIS) was used in this retrospective study. The NIS is a large publicly available database created and distributed by the Healthcare Cost and Utilization Project (HCUP) to encourage research in cost reduction and quality improvement [11]. The NIS contains 20% of all inpatient hospitalizations in the United States and, when unweighted, contains information on eight million annual hospital admissions.

Patient selection

The NIS was queried from January 1, 2009 to December 31, 2016 using International Classification of Disease, 9th and 10th revision (ICD-9 and -10) diagnosis codes to identify all primary THA admissions, excluding revision THA patients. A total of 2,676,086 patients were identified and categorized as non-obese (n=2,222,836), overweight (n=21,222), non-morbidly obese (n=298,360), and morbidly obese (n=133,585).

Variables analyzed

Patient demographics included age, sex, race (white, black, Hispanic, Asian, native American, other race), median household income by quartile, and primary payer (Medicare, Medicaid, private insurance, self-pay, no charge, other pay). Patient health status was classified using the age-adjusted Charlson Comorbidity Index (CCI), a predictive tool that estimates one-year mortality based on the presence of 19 comorbidities, which was calculated using ICD-9 and -10 diagnosis codes [12]. Patient outcomes included length of stay (LOS), mortality, discharge disposition (routine home, short-term hospital, other facility, home healthcare, left against medical advice, died, and unknown), and postoperative complications. Total hospital charges, defined as the amount billed to the payer for the inpatient stay, is a data element in the NIS. However, hospital cost must be estimated from charges utilizing the “cost-to-charge ratio” supplemental file provided by HCUP [11]. Costs and charges were adjusted for inflation to January 2019 dollars according to the consumer price index [13].

Statistics

Differences in demographics and outcomes between non-obese, overweight, non-morbidly obese, and morbidly obese patients were compared. Categorical variables were evaluated with chi-square analyses, while continuous variables were evaluated with one-way analyses of variance. A p-value of 0.050 or less was set as the threshold for statistical significance. All statistical analyses were conducted with SPSS version 25 (IBM Corp., Armonk, NY).

## Results

Incidence

**Figure 1:**
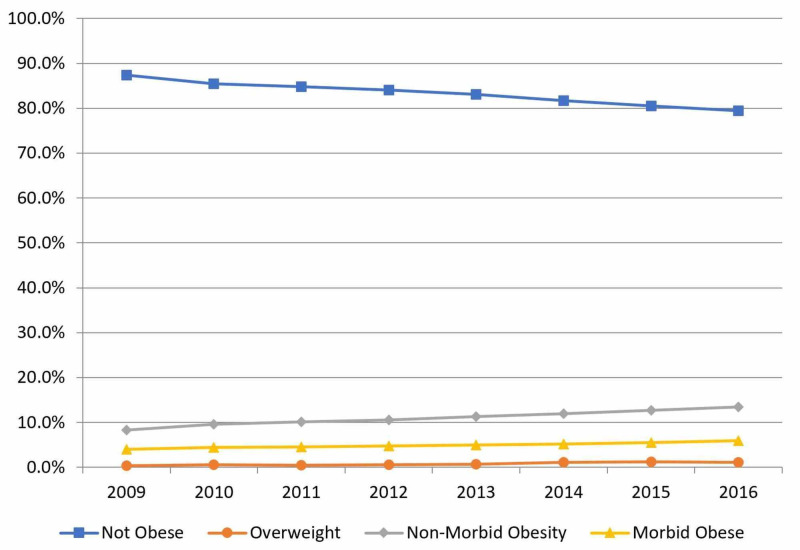
Obesity Classifications in Total Hip Arthroplasty by Year

Between 2009 and 2016, non-obese, overweight, non-morbidly obese, and morbidly obese patients comprised 83.1%, 0.8%, 11.2%, and 5.0% of all THAs, respectively (Figure 1). The rate of increase in THA utilization was 299.0% for overweight, 109.3% for non-morbidly obese, and 90.9% for morbidly obese patients. When evaluated by proportions, non-obese patients decreased by 7.84% while overweight, non-morbidly obese, and morbidly obese patients increased by 0.76%, 5.14%, and 1.94%, respectively.

Patient demographics

Non-obese, overweight, non-morbidly obese, and morbidly obese patients had an average age of 66, 64, 63, and 61 years, respectively (p<0.001) (Table 1). Compared with non-obese patients, there were significantly less overweight and more morbidly obese patients who were female (55.6% non-obese vs 50.9% overweight vs 54.7% non-morbidly obese vs 60.4% morbidly obese, p<0.001). There was a significantly greater proportion of white patients who were non-obese when compared with other BMI categories (86.6% vs 85.7% vs 84.0% vs 81.5%, respectively), while morbid obesity demonstrated the highest proportion of black patients (7.0% vs 7.0% vs 9.4% vs 12.3%, respectively; both p<0.001). Significantly more morbidly obese patients lived in the lowest income areas (19.7% vs 15.6% vs 19.8% vs 22.7%, respectively), while less lived in the highest income areas (28.7% vs 39.0% vs 27.2% vs 23.3%, respectively; p<0.001). Medicare was the primary payer for all groups except the morbidly obese patients (53.8% vs 46.4% vs 45.3% vs 41.5%, respectively). In contrast, morbidly obese patients were the highest users of Medicaid (4.0% vs 3.3% vs 4.5% vs 6.4%, respectively) and private insurance (38.8% vs 48.0% vs 46.7% vs 48.7%, respectively; both p<0.001). Finally, morbidly obese patients demonstrated the lowest proportion of patients with a CCI of 3 or more (74.4% vs 70.1% vs 71.1% vs 68.5%, respectively; p<0.001).

**Table 1:** Patient Demographics N: number; SD: standard deviation.

Parameter (N) (%)	Non-obese (N=2,222,836)	Overweight (N=21,222)	Non-morbidly obese (N=298,360)	Morbidly obese (N=133,585)	P-value
Mean age (years) (SD)	66 (12)	64 (11)	63 (11)	61 (10)	<0.001
Sex					
Male	197,789 (44.4%)	2,087 (49.0%)	135,178 (45.3%)	52,895 (39.6%)	
Female	248,056 (55.6%)	10,812 (50.9%)	163,182 (54.7%)	80,690 (60.4%)	<0.001
Race					
White	352,747 (86.6%)	16,940 (85.7%)	227,906 (84.0%)	99,316 (81.5%)	
Black	28,467 (7.0%)	1,380 (7.0%)	25,572 (9.4%)	15,017 (12.3%)	
Hispanic	13,115 (3.2%)	657 (3.3%)	10,366 (3.8%)	4,392 (3.6%)	
Asian	3,868 (0.9%)	168 (0.8%)	1,363 (0.5%)	389 (0.3%)	
Native	1,397 (0.3%)	45 (0.2%)	865 (0.3%)	394 (0.3%)	
Other race	7,936 (1.9%)	586 (3.0%)	5,209 (1.9%)	2,302 (1.9%)	<0.001
Median household income					
Quartile 1	86,580 (19.7%)	3,246 (15.6%)	58,240 (19.8%)	29,873 (22.7%)	
Quartile 2	109,116 (24.9%)	4,460 (21.5%)	73,799 (25.1%)	34,739 (26.4%)	
Quartile 3	117,062 (26.7%)	4,972 (23.9%)	81,856 (27.9%)	36,188 (27.5%)	
Quartile 4	125,723 (28.7%)	8,098 (39.0%)	79,913 (27.2%)	30,682 (23.3%)	<0.001
Primary payer					
Medicare	239,883 (53.8%)	9,829 (46.4%)	134,809 (45.3%)	55,352 (41.5%)	
Medicaid	17,920 (4.0%)	707 (3.3%)	13,542 (4.5%)	8,550 (6.4%)	
Private	172,884 (38.8%)	10,160 (48.0%)	139,269 (46.7%)	64,984 (48.7%)	
Self-pay	3,425 (0.8%)	117 (0.6%)	2,027 (0.7%)	958 (0.7%)	
No charge	549 (0.1%)	11 (0.1%)	379 (0.1%)	175 (0.1%)	
Other pay	11,034 (2.5%)	353 (1.7%)	7,883 (2.6%)	3,348 (2.5%)	<0.001
Charlson Comorbidity Index					
0	8,950 (2.0%)	455 (2.1%)	4,868 (1.6%)	2,681 (2.0%)	
1	26,702 (6.0%)	1,249 (5.9%)	21,002 (7.0%)	11,114 (8.3%)	
2	78,691 (17.6%)	4,632 (21.8%)	60,475 (20.3%)	28,342 (21.2%)	
3+	332,076 (74.4%)	14,886 (70.1%)	212,064 (71.1%)	91,481 (68.5%)	<0.001

Patient outcomes

Overweight patients had the shortest LOS (2.67 days), while morbidly obese patients had the longest LOS (3.18 days; p<0.001) (Table 2). Morbidly obese patients comprised the lowest proportion of routine (28.7% non-obese vs 28.9% overweight vs 30.2% non-morbidly obese vs 26.0% morbidly obese) and home health (41.4% vs 49.2% vs 42.2% vs 39.3%, respectively) discharges, but the highest percentage of other facility discharges (28.9% vs 21.4% vs 26.9% vs 34.0%, respectively; all p<0.001). Morbidly obese patients experienced the highest proportion of postoperative inpatient complications (28.8% vs 25.3% vs 28.0% vs 30.8%, respectively; p<0.001). The mortality rate was significantly, albeit not clinically, different between categories.

**Table 2:** Patient Outcomes N: number; SD: standard deviation. **In accordance with Healthcare Cost and Utilization Project reporting guidelines, cells with less than 11 patients were masked.

Parameter (N) (%)	Non-obese (N=2,222,836)	Overweight (N=21,222)	Non-morbidly obese (N=298,360)	Morbidly obese (N=133,585)	P-value
Mean length of stay (days) (mean [SD])	3.01 (2.15)	2.67 (1.49)	2.87 (1.81)	3.18 (2.59)	<0.001
Mortality					
Alive	445,606 (99.9)	21,198 (100.0%)	298,016 (99.9%)	133,441 (99.9%)	
Died	587 (0.1)	** (0.0%)	195 (0.1%)	99 (0.1%)	0.002
Discharge disposition					
Routine	128,158 (28.7%)	6,133 (28.9%)	90,017 (30.2%)	34,670 (26.0%)	
Short-term hospital	3,213 (0.7%)	94 (0.4%)	1,582 (0.5%)	778 (0.6%)	
Other facility	129,010 (28.9%)	4,527 (21.4%)	80,347 (26.9%)	45,394 (34.0%)	
Home healthcare	185,014 (41.4%)	10,439 (49.2%)	125,915 (42.2%)	52,534 (39.3%)	
Left against medical advice	190 (0.0%)	** (0.0%)	156 (0.1%)	55 (0.0%)	
Died	587 (0.1%)	** (0.0%)	195 (0.1%)	99 (0.1%)	
Unknown	21 (0.0%)	** (0.0%)	** (0.0%)	** (0.0%)	<0.001
Complications					
No complications	317,744 (71.2%)	15,850 (74.7%)	214,974 (72.0%)	92,497 (69.2%)	
Complications present	128,675 (28.8%)	5,728 (25.3%)	90,537 (28.0%)	45,413 (30.8%)	<0.001
Charges (mean [SD])	$62,776 ($38,027)	$64,189 ($29,949)	$62,678 ($36,225)	$65,601 ($43,840)	<0.001
Costs (mean [SD])	$18,282 ($8,868)	$19,767 ($7,891)	$18,477 ($8,820)	$19,105 ($9,427)	<0.001

Charges and cost

Charges were greatest for morbidly obese patients ($62,776 non-obese vs $64,189 overweight vs $62,678 non-morbidly obese vs $65,601 morbidly obese; p<0.001), while cost was highest for overweight patients ($18,282 vs $19,767 vs $18,477 vs $19,105, respectively; p<0.001).

## Discussion

Since obesity now affects one-third of the American population, the inevitable rise in coxarthrosis may lead to an exponential increase in the demand for THA by morbidly obese patients [1,14]. However, some surgeons and health systems restrict THA in morbidly obese patients to avoid the extra associated cost and complications. This may alienate populations that already face disparities in access to care, such as those of lower socioeconomic status and less represented ethnicities such as black and Hispanic populations. This study compared various BMI categories to assess for differences in demographics and outcomes. The results demonstrated a proportional increase in all BMI categories above 25 kg/m^2^. Specifically, the highest proportions of female, black, low-income, Medicaid, and private insurance patients were morbidly obese. Additionally, morbidly obese patients demonstrated the longest LOS, the most complications, the highest charges, and the second highest costs with the lowest proportion of routine home discharges. This represents a potential crisis in access to care if hospitals refuse morbidly obese patients to prevent complications and reduce cost, as morbidly obese patients are comprised of a higher proportion of minority and low-income populations. 

This study is not without limitations. Foremost, the NIS is an administrative database that is limited to the data elements collected. However, the database contains many important variables with substantial research potential. Furthermore, the NIS draws information only from the inpatient stay; thus, the patient cannot be followed longitudinally through their post-discharge course. Nevertheless, since this database is useful for research on LOS, demographics, cost, and trends, it is appropriate for the present study. Finally, the time period researched in the present study spans the transition from ICD-9 to ICD-10 diagnosis codes. Since ICD-10 offers a greater variety and detail of diagnosis codes than its predecessor, there is potential for coding errors as surgeons and hospital systems adjust to the change. Still, most of these errors would have been caught by HCUP, as the NIS undergoes extensive quality control. Despite these limitations, this study has value as it reports on important trends in morbid obesity in THA with a large sample size. 

Previous studies have documented the increasing incidence of obesity in THA, such as the 2019 retrospective review by Pirruccio et al. [15]. This study analyzed 135,013 THAs from the National Surgical Quality Improvement Program (NSQIP) database between 2008 and 2016. They reported an increase in the average BMI from 30.18 to 30.26 kg/m^2^ in that timespan, and a proportional increase in the prevalence of obesity of 1.64%. Additionally, they reported an association between morbid obesity and a decreased rate of discharge home (odds ratio [OR]=0.73, p<0.001) and increased LOS (OR=1.19, p=0.004). The present study observed a greater increase in non-morbid (5.14%) and morbidly obese (1.94%) patients, while also reporting morbid obesity having the lowest proportion of patients discharged home and the highest LOS. The differences in incidences between studies may be due to the nature of the NIS and the NSQIP, as the NIS pulls information from a greater number of hospitals and thus, may be more representative of national estimates.

Morbidly obese patients were observed to have the highest complication rate compared to other BMI categories. These results are similar to previous research by Deakin et al., who reported more complications (p=0.047) in morbidly obese patients than in non-obese patients in a single institutional study of 906 THAs [16]. Additionally, the present study observed a higher cost of THA in morbid obese patients compared to non-obese patients. This supports another NIS study by Kim et al., who reported a 9% increase in the cost of morbidly obese patients undergoing THA compared to non-obese patients [17]. Even though complications and costs remain higher for morbidly obese patients, a cost-effectiveness study by Ponnusamy et al. suggests that the long-term benefits of THA on patients of all BMI categories outweigh the short-term risks and costs [10]. Thus, the practice of denying patients access to THA due to their BMI may be doing more harm to society than good. 

The present study observed the highest proportion of black patients among the morbidly obese category. This reflects the current obesity epidemic, which affects black and Hispanic Americans at higher rates than any other ethnic group according to a retrospective review of a large administrative database which followed obesity trends between 1999 and 2016 by Skinner et al. [18]. Additionally, in a prospective cohort study of 568 patients at a single institution, Brock et al. reported that higher BMI was associated with a younger age of total joint arthroplasty [19]. Furthermore, higher BMI patients were less likely to be non-Hispanic whites. Ostensibly, there may be an increasing demand for THA among morbidly obese patients who are younger and more diverse. Therefore, any decision to deny patients' THA based on modifiable risk factors should be made with cognizance not to ostracize patient populations experiencing existing disparities in care. 

## Conclusions

The results of this study reflect the ongoing obesity epidemic, as the proportion of morbidly obese patients undergoing THA increased significantly. Furthermore, morbidly obese patients demonstrated higher rates on complications, longer LOS, and higher cost than non-obese patients. Morbid obesity also demonstrated the highest proportion of black patients compared to other groups. Since BMI is considered a modifiable risk factor, preoperative optimization of weight may improve clinical and economic outcomes. However, as the incidence of morbid obesity continues to grow, further research may be needed to determine whether the efforts to avoid THA on morbidly obese patients further harms populations who already experience disparities in care. 
